# On the importance of membership organizations to the governmental public health workforce, through good times and bad

**DOI:** 10.1093/pubmed/fdac098

**Published:** 2022-11-21

**Authors:** Jonathon P Leider

**Affiliations:** Division of Health Policy and Management, Center for Public Health Systems, University of Minnesota School of Public Health, Minneapolis, MN, USA

It is often an expectation that institutions endure, but it is not a given. That is why, on occasions like the 50th anniversary of the UK Faculty of Public Health, it is worth reflecting on the importance of the role organizations like these play in the broader system. The governmental public health workforce across the globe looks exceedingly different, is organized exceedingly differently and exists for different reasons.^1–3^ As Czabanowska and Middleton highlight, however, commonalities of need exist internationally around professionalism, shortages and the fundamental need to define what public health workers are and what they do.^1^ This is one of the primary roles and functions of membership organizations.

Membership groups, associations and organizations self-structure around disciplines, roles and shared interests to further those interests. They set standards, provide and assure training, and can be one of the primary bodies in advocating for a decent minimum of knowledge, skills and abilities for the public health workforce in their country, state or province. One might argue, also, that they can be a primary bulwark for the workforce against the pressures and tides of the day.

## The American context, politics, austerity and the role of membership organizations

As Krasna elucidates in her commentary in this special issue, the American response to public health is fractious and fractured.^4^ In some ways, public health is likely past its zenith of influence from 2020/2021 where COVID, vaccines and lockdowns made public health a household name. But even if, nominally, the notion of public health is waning from the collective consciousness of the American public, it represents a substantial opportunity for impact relative to its position prior to the pandemic, where public health had often been referred to as ‘invisible’—omnipresent, but unknown to the public.^5–7^

The second, more pragmatic reason for opportunity is a truth of government: public dollars move slower than public sentiment. As part of COVID-19 response and the Biden Administration’s budget priorities, over $7 billion have been allocated toward public health workforce.^8^ Though historic, this is one-time money. This is perhaps more money than the public health system has ever seen at one time, and could be more money than we know how to spend; it may well be subject to rescission, otherwise reverted or reclaimed if unspent, and leave politicians wondering about investing so much in the future. Thus, a reasonable fear is—what comes next?

There is inevitably backlash and movement toward austerity after enormous federal or national investment. Membership organizations represent the very staff facing this backlash; in the United States, there are dozens of public health-oriented membership associations and organizations, of varying sizes, scopes and missions. Whether in the United States or other countries, it is incumbent on national and state systems to evaluate and demonstrate value in public health spending in systems to make the case that investments were worthwhile. This space is one of the most important membership organizations occupy—educating, and informing, if not advocating and lobbying.

Membership organizations must see the ‘writing on the wall,’ that backlash is coming, the funding cliff is ahead of them and begin to prepare now for those conversations that will happen in 3–5 years. In years past, austerity in the US led to deep cuts to the workforce that we had not properly recovered from, pre-COVID; this hampered the nation’s COVID-19 response.^4^ Although cuts may not be completely avoided, but anticipation now of a demand for return-on-investment demonstrations later may abridge later deeper cuts.

## A time for professionalism, standard-setting and investment in the workforce

There is time, money and interest to grow and standardize the public health workforce.^1^ Historic public health workforce shortages, burnout and turnover will complicate response and recovery efforts. In the United States, at least 80 000 full-time equivalents are needed to deliver Foundational Public Health Services.^9^ Almost half (44%) of the state and local workforce recently reported considering leaving or planning to retire in the next 5 years.^10^ A quarter of the workforce report three or more symptoms of post-traumatic stress syndrome, and 41% of public health executives say they have been bullied or harassed.^10^

It is a challenging time to be a public health worker.

Being an advocate for the public health workforce does not mean merely advocating for increased number of employees. In an increasingly complex and politicized environment, staff face mental health challenges and other needs. Membership organizations, in representing their members, play a critical role in communicating to policymakers the needs of all public health staff. It is incumbent on all public health membership organizations—old and new, small or large, to elevate the status of public health practitioners, to move toward standardization in their home countries and to seek sustained and sustainable funding streams to build back the workforce from a once-in-a-generation pandemic response and recovery effort.
